# Sexual Transmission of Zika Virus: An Assessment of the Evidence

**Published:** 2017-09

**Authors:** Mohammad ZAMANI, Vahid ZAMANI

**Affiliations:** 1. Student Research Committee, Babol University of Medical Sciences, Babol, Iran; 2. Cancer Research Center, Babol University of Medical Sciences, Babol, Iran; 3. Vice-Chancellery for Health, Babol University of Medical Sciences, Babol, Iran

## Dear Editor-in-Chief

Zkia virus (ZIKV) is a flavivirus from the flaviviridae family, transmitted to humans predominantly by infected mosquitoes (mostly by *Aedes* spp.). This arbovirus was initially isolated from a rhesus monkey in the Zika forest in Uganda in 1947. ZIKV infection is commonly associated with unclear symptoms. However, in about 20% of cases, it can cause self-limited symptoms such as fever, joint pain, rash, and conjunctivitis for several days (1). ZIKV likely causes microcephaly in babies, suggesting the congenital and perinatal transmission. Another concern is about the relation between ZIKV and Guillain-Barré syndrome (1).

Although the principal route of transmission of ZIKV is through mosquitoes, the sexual transmission of ZIKV is possible. There are a few cases in this regard: 1) probability of the sexual transmission of a male patient was infected in southeastern Senegal in 2008, to his wife (2). Four days after returning the patient to his home in Colorado and United States; symptoms of ZIKV infection appeared in his wife. She had not left the United States since previous year and had a vaginal sex with her husband one day after his return to home. That is why; the authors suggested the transmission by infected semen. 2) In French Polynesia, ZIKV RNA was detected in a patient’s semen who presented with hematospermia (after third episode of the infection, approximately 10 wk after disease onset), although ZIKV was not isolated from the blood samples taken at the same time (3). 3) On Feb 2016, the Dallas County Health and Human Services reported a case who acquired the ZIKV through sex with a patient who returned from Venezuela where ZIKV infection is spreading (4). 4) In Paris, France, infection symptoms were observed in a woman in Feb 20, 2016 (5). She had not any travels outside France since Jan 2016, and was not exposed to any risk factors, like receiving blood transfusions, but had a sexual contact between Feb 11 and Feb 20, 2016, with a male patient who was in Brazil a few days ago. 5) A woman was represented suspected of being infected through a delayed sexual transmission (6). She and her partner traveled to Martinique (an island in the Caribbean Sea). Before they returned to France on Feb 7, 2016, the man displayed the symptoms. His infection was confirmed by serology 53 d after the beginning of the presentation. The couple had several sexual intercourses after their return. Forty days later, the infection appeared to the woman. In addition to these, during Jan to Apr 2016, nine cases from the United States reported being sexually infected through male-to-female transmission (7).

The isolation of the infectious virus from semen and saliva (5, 8), and RNA of the virus has been detectable from semen until 62 d post-symptom onset (1). Moreover, the sexual transmission was recently declared in a mouse model, that is, ZIKV was sexually transmitted from male mice to female mice. Viral RNA persisted in semen for 58 d post-inoculation (9).

ZIKV can be transmitted from infected men to their sex partners through vaginal, anal, and oral sex; however, sexual transmission from women to others has not been stated (10). Altogether, use of condom, use of safer sexual practices and contraceptive measures are necessary for persons living in infected areas, or those who traveled there (up to 1 month after their return).
